# Primary Osteocyte Supernatants Metabolomic Profiling of Two Transgenic Mice With Connexin43 Dominant Negative Mutants

**DOI:** 10.3389/fendo.2021.649994

**Published:** 2021-05-18

**Authors:** Meng Chen, Guobin Li, Lan Zhang, Kaiting Ning, Baoqiang Yang, Jean X. Jiang, Dong-En Wang, Huiyun Xu

**Affiliations:** ^1^ Key Laboratory for Space Bioscience and Biotechnology, School of Life Sciences, Northwestern Polytechnical University, Xi’an, China; ^2^ Department of Biochemistry and Structural Biology, University of Texas Health Science Center, San Antonio, TX, United States; ^3^ Research Center of Special Environmental Biomechanics & Medical Engineering, Northwestern Polytechnical University, Xi’an, China

**Keywords:** connexin43, osteocyte, hemichannel, metabolomics, metabolite

## Abstract

Osteocytes could release some small molecules (≤ 1 kDa) through gap junctions and hemichannels to extracellular environment, such as prostaglandin E2 (PGE2), nitric oxide (NO) and adenosine triphosphate (ATP), which play key roles in transferring signals between bone cells and other tissue cells. Connexin (Cx) 43 is the most abundant connexin in osteocytes. To further discover molecules released by osteocytes through Cx43 channels and better understand the regulatory function of Cx43 channels in osteocytes, we performed non-targeted global metabolomics analysis using liquid chromatography-tandem mass spectrometry (LC-MS/MS) on conditioned medium collected from osteocytes isolated from two transgenic mouse models with Cx43 dominant negative mutants driven by a 10 kb-DMP1 promoter: R76W (gap junctions are blocked, whereas hemichannels are promoted) and Δ130-136 (both gap junctions and hemichannels are blocked). The results revealed that several new categories of molecules, such as “fatty acyls” and “carboxylic acids and derivatives”, could be released through osteocytic Cx43 channels. In addition, alteration of Cx43 channel function affected the release of metabolites related to inflammatory reaction and oxidative stress. Pathway analysis further showed that citric acid cycle was the most differential metabolic pathway regulated by Cx43 channels. In sum, these results isolated new potential metabolites released by osteocytes through Cx43 channels, and offered a novel perspective to understand the regulatory mechanisms of osteocytes on themselves and other cells as well.

## Introduction

The osteocyte is the most abundant cell type (90% - 95%) in bone tissue (1). It is well known that osteocytes are completely embedded in the bone matrix and form a complex network of interconnecting lacuna-canaliculi, which achieves direct communication between osteocytes and other cell types, including osteoblasts, osteoclasts, mesenchymal stromal cells, and neurocytes (2). More importantly, osteocytes are regarded as the source of various signaling molecules such as sclerostin, fibroblast growth factor-23 (FGF23), Dickkopf-related protein 1 (DKK1), and vascular endothelial growth factor (VEGF), which could target adjacent or distant organs (3–6). Furthermore, connexin 43 (Cx43), the most abundant connexin expressed in osteocytes (7), forms gap junctions and hemichannels that serve as physical linkages to allow the exchange of small molecules (≤ 1 kDa) between adjacent cells and extracellular environment (8). Hence, gap junctions and hemichannels play pivotal roles in regulating signal transduction not only between osteocytes and other bone cells to participate in the modeling and remodeling of the bone (9), but also between osteocytes and other tissue cells to regulate the function of muscle, kidney, and fat metabolism (10–12).

It has previously been shown that some small molecules (PGE2, NO, and ATP) are released into the extracellular environment by osteocytes *via* Cx43 channels (13, 14), which impact diverse cellular signaling pathways including Wnt/β-catenin, receptor activator of nuclear factor κB ligand/osteoprotegerin (RANKL/OPG), and calcium signaling pathways (15–17). However, more molecules are still not clear and needed to be discovered, which are essential to further understand the molecular mechanisms underlying osteocyte-mediated effects on bone homeostasis and other systemic diseases.

Metabolomics is a powerful and comprehensive tool, providing quantitative and qualitative analysis of low-molecular-mass metabolites (≤ 1 kDa) in biological matrices (18). The size of molecules released from Cx43 channels is usually smaller than 1 kDa, therefore, metabolomics analysis is a suitable technique to investigate the molecule profiles.

In the current study, two transgenic mouse models were used, each carrying Cx43 dominant negative mutants driven by a 10 kb-DMP1 promoter: R76W and Δ130-136. Gap junction channel function is blocked in R76W mice, whereas hemichannels are specifically elevated. In Δ130-136 mice, both gap junction and hemichannel channels are inhibited (19). The conditioned medium of primary osteocytes was collected from wild-type (WT), R76W and Δ130-136 mice for metabolomic analysis of molecules in the ≤ 1 kDa fraction. The differential metabolites obtained will give some novel clues to study the regulatory function of osteocytes on other cells, and provide some potential targets for treatment of related diseases.

## Materials and Methods

### Animals

Two transgenic mouse models with Cx43 dominant negative mutants in osteocytes, R76W and Δ130-136, were generated at the University of Texas Health Science Center at San Antonio (UTHSCSA). For R76W mice, gap junction channels are blocked, whereas hemichannels are specifically elevated. However, for Δ130-136 mice, both of gap junction and hemichannel channels are inhibited. Mice were housed under specific pathogen-free conditions at 25°C, 40% relative air humidity with a 12-hour alternating light/dark cycle and free access to water and food. Genotyping was performed by real-time PCR using genomic DNA isolated from mouse toe. All animal protocols were approved by the Northwestern Polytechnical University (NPU) Institutional Animal Care and Use Committee.

### Isolation of Primary Osteocytes From Mice

The isolation and culture of primary osteocytes were based on a published protocol (20). Briefly, three-month-old male R76W, Δ130-136 and WT C57BL/6J mice were sacrificed and long bones were collected. Muscles around long bones were removed with sterile gauze. Both ends of long bones were cut off and bone marrow was flushed using PBS (phosphate buffer saline). Long bones were cut into pieces ranging from 1 to 2 mm in length and washed twice with D-Hank’s solution. The bones were digested with collagenase type I (Gibco, USA) and EDTA (5 mM) on the shaker in a 5% CO_2_ incubator at 37°C. After multiple digestions, the final digests containing enriched osteocytes were collected. 1 × 10^6^ isolated osteocytes were plated on a 35 mm collagen I-coated dish and cultured in 3 mL α-MEM (Gibco, USA) with 5% fetal bovine serum (FBS, Hyclone, USA), 5% bovine calf serum (BCS, Hyclone, USA) and 1% penicillin-streptomycin solution (Beyotime, China). The cells were kept in an incubator (5% CO_2_) at 37°C for 5 days.

### Conditioned Medium Collection and Metabolite Extraction

Supernatants were collected after 5 days of culture. Supernatants were stored at -80°C and thawed at 4°C for metabolite extraction. 100μL of supernatant was treated with 300μL precooled methanol. The mixture was precipitated at -20°C for 2 h, followed by centrifugation at 30,000 g, 4°C for 20 min. 290μL of the supernatant was transferred to a new eppendorf tube prior to liquid chromatography separation.

### UPLC-MS/MS Analysis

All samples were acquired by the LC-MS system. All chromatographic separations were performed using a 2777C ultra performance liquid chromatography (UPLC) system (Waters, U.K.). An ACQUITY UPLC HSS T3 column (100mm × 2.1mm, 1.8μm, Waters, U.K.) was employed for the reversed phase separation. The column oven was kept at 50°C and the flow rate was 0.4 mL/min. The mobile phase comprised solvent A (water and 0.1% formic acid) and solvent B (acetonitrile and 0.1% formic acid). Gradient elution conditions were set as follows: 100% phase A, 0-2 min; 0% to 100% phase B, 2-11 min; 100% phase B, 11-13 min; 0% to 100% phase A, 13-15 min. The injection volume for each sample was 10 μL. Next, a high-resolution tandem mass spectrometer Xevo G2-XS QTOF (Waters, UK) was used to detect metabolites eluted from the column in both positive and negative ion modes. For positive and negative ion mode, the capillary and sampling cone voltages were set at 3.0 kV and 40.0 V, 2.0 kV and 40.0 V, respectively. Furthermore, the mass spectrometry data were obtained in Centroid MSE mode. The TOF mass range was from 50 to 1200 Da and the scan time was 0.2 s. Finally, for the MS/MS detection, the whole precursors were fragmented using 20-40 eV, and the scan time was 0.2 s. The LE signal was acquired every 3s to calibrate the mass accuracy. To assess the stability, reliability and reproducibility of the LC-MS/MS system, quality control (QC) samples were obtained by mixing equal volumes (40μL) from each tested sample and employed during the whole acquisition. Furthermore, a QC sample (Pool of all samples) was acquired after every 10 tested samples.

### Data Processing and Analysis

Raw data files were uploaded into Progenesis QI software (version 2.2, Waters, UK), whose workflow mainly included peak alignment, peak picking and peak identification. MetaX software was used to perform pre-processing of the extracted data (21), including removing low-quality ions [relative standard deviation (RSD) in the QC samples > 30%]. After quality control-based robust LOESS signal correction (QC-RLSC), the data matrix was analyzed by both principal component analysis (PCA) and partial least squares discriminant analysis (PLS-DA) and a 95% confidence interval (CI) was used as the threshold. As noted, QC-RLSC is an effective method of data correction in the field of metabolomics (22). PCA is a technique of multivariate statistical analysis for reducing the dimensionality of the data, increasing interpretability but retaining most of the variation in the data set (23). PLS-DA is a data analysis tool for classification to reflect the differences between random groups (24). They are both commonly used in metabolomics analysis. The quality of the models was evaluated with the relevant R2 and Q2 as previously reported (25). Meanwhile, permutation tests (200 cycles) were implemented to evaluate the robustness of the PLS-DA model. When the variable influence on projection (VIP) values from the PLS-DA model were greater than 1.0, differential metabolites were selected. Statistically significant differences were tested using Student’s t-test and fold change (FC) analysis, and the p-value was corrected into q-value by false discovery rate (FDR). Generally, metabolites with the characteristics of VIP ≥ 1, FC ≥ 1.2 or ≤ 0.8 and q-value < 0.05 were regarded as significantly difference between the mouse groups.

Based on the abundance of differential metabolites, heatmaps were generated using the Multiple Experiment Viewer (MeV) software (http://mev.tm4.org/). Meanwhile, we utilized Kyoto Encyclopedia of Genes and Genomes (KEGG; https://www.kegg.jp) and Human Metabolome Database (HMDB; http://www.hmdb.ca) to check and confirm the putative differential metabolites. Candidate metabolites were determined by MS/MS scans for the characteristic ions and fragmentation patterns of the compound. Pathway and enrichment analysis were performed using MetaboAnalyst 4.0 (http://www.metaboanalyst.ca), which is a powerful web tool for statistical, functional and integrative analysis of metabolomics data. In the MetaboAnalyst analysis, little p-value and large pathway impact factor indicate that the pathway is highly affected.

### Statistical Analysis

Statistical analysis was performed using GraphPad Prism7 statistics software (GraphPad). Data were presented as mean ± SD. Comparisons between two groups were analyzed using 2-tailed unpaired Student’s t-tests. A value of *P* < 0.05 was considered statistically significant.

## Results

### Metabolic Profiles of Primary Osteocyte Supernatants

Previously, we have found that Δ130-136 or R76W in transgenic mice exhibited dominant negative effects on gap junction channels and hemichannels or only on gap junction channels, respectively (19).

**Figure 1:**
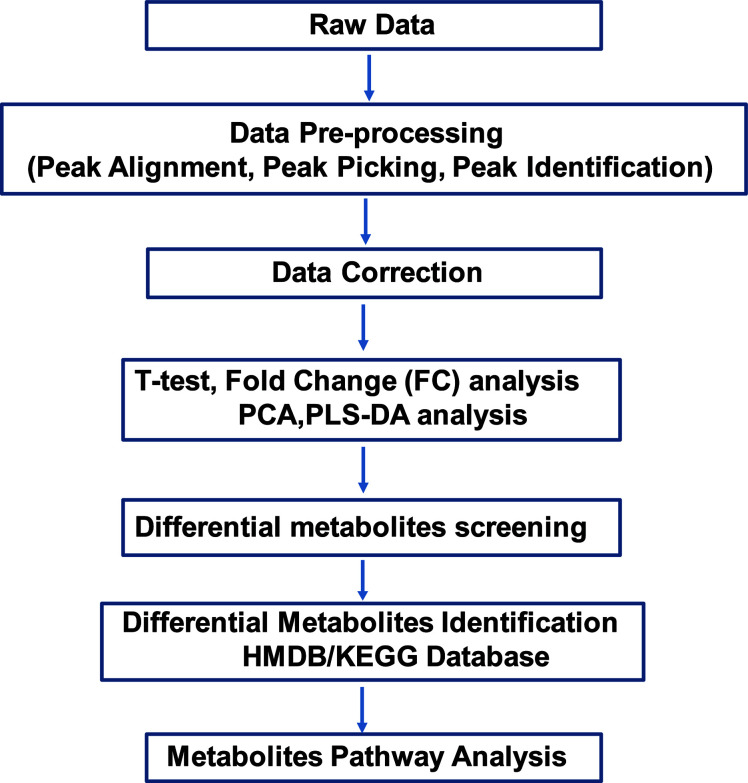
Overview of the study. Non-targeted metabolomics was performed to discover potential metabolites in primary osteocyte supernatants. Raw data files were uploaded to conduct pre-processing, including peak alignment, peak picking and peak identification. After data correction, the data matrix was analyzed by both univariate analysis (T-test and FC analysis) and multivariate analysis (PCA and PLS-DA). Next, screening and identifying the differential metabolites were conducted by searching against the mass-based HMDB/KEGG database. Finally, pathway analysis of the differential metabolites was applied among WT, R76W and Δ130-136 mouse groups. FC, fold change; PCA, principal component analysis; PLS-DA, partial least squares discriminant analysis.

**Figure 2:**
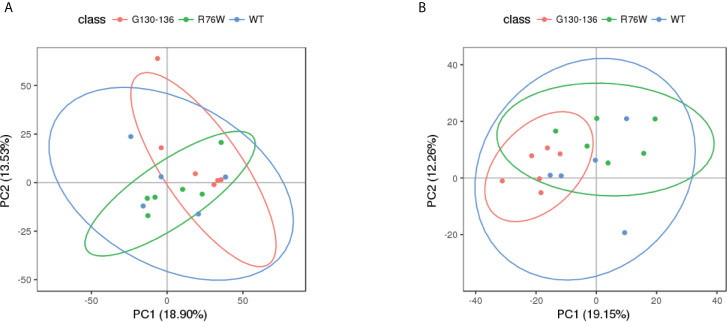
PCA score scatter plots of tested samples. PCA score scatter plots in ESI+ mode **(A)** and ESI− mode **(B)**. WT (blue), n=5; R76W (green), n=6; Δ130-136 (red), n=6.

**Figure 3:**
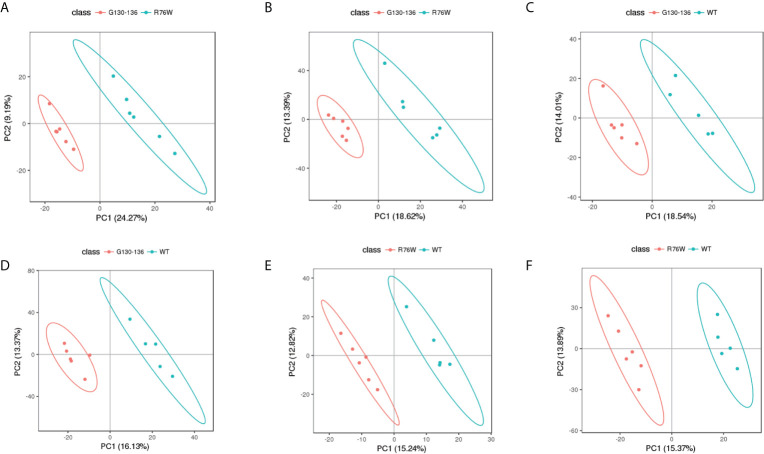
PLS-DA score scatter plots of tested samples. PLS-DA score scatter plots of Δ130-136 versus R76W in ESI+ mode **(A)** and ESI− mode **(B)**. Δ130-136 (red) and R76W (blue). PLS-DA score scatter plots of Δ130-136 versus WT in ESI+ mode **(C)** and ESI− mode **(D)**. Δ130-136 (red) and WT (blue). PLS-DA score scatter plots of R76W versus WT in ESI+ mode **(E)** and ESI− mode **(F)**. R76W (red) and WT (blue).

Non-targeted metabolomics analysis was performed on the supernatants of primary osteocytes isolated from WT, R76W and Δ130-136 mice. **Figure 1** illustrates the detailed workflow of the metabolomics analysis. To evaluate the capability of the LC-MS/MS-based metabolomics approach performed in the study, we first analyzed the total ion chromatograms of quality control (QC) samples. As shown in **Figure S1**, there was stable retention time and no obvious peak drift in total ion chromatograms. 8,866 and 4,526 total ions were identified in ESI+ and ESI− mode, respectively. After removing low-quality ions [relative standard deviation (RSD) > 30%], 6,878 and 3,417 ions were identified in ESI+ and ESI− mode, respectively. Nine QC samples were employed for tested samples throughout the entire analysis. As shown in **Figure S2**, principal component analysis (PCA) among the QC and tested samples exhibited that QC samples were gathered together and separated from the tested samples, thereby indicating the stability, reliability and reproducibility of the LC-MS/MS analysis. To better clarify the metabolic variations in the supernatants of primary osteocytes of WT, R76W and Δ130-136 mice, PCA and partial least-squares discriminant analysis (PLS-DA) were performed to process the data. PCA score plots in ESI+ mode and ESI− mode showed that samples of each group were clustered together (**Figures 2A, B**), which manifested small differences and good parallelism within each group. Furthermore, PLS-DA models showed apparent separation between random two groups in ESI+ mode and ESI− mode (**Figures 3A–F**), indicating a significant difference between random two groups.

### Metabolic Variations Among Groups of WT, R76W and Δ130-136 Mice

**Figure 4:**
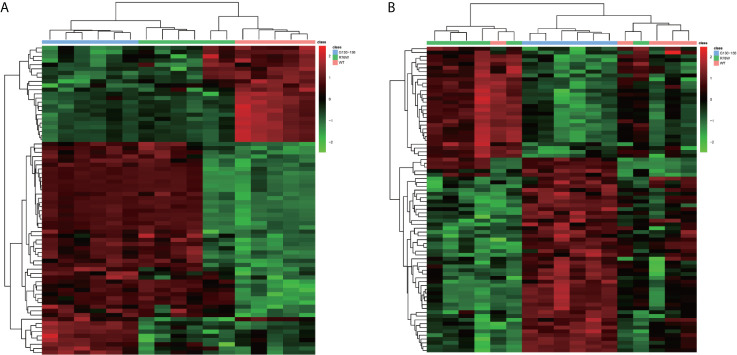
Comparison of primary osteocyte supernatant metabolites among Δ130-136, R76W and WT mouse groups. **(A)** Heat map of 74 differential metabolites among three mouse groups in ESI+ mode. **(B)** Heat map of 80 differential metabolites among three mouse groups in ESI− mode. WT (red), R76W (green) and Δ130-136 (blue).

In order to visually distinguish the differences in metabolites among WT, R76W and Δ130-136 mouse groups, heat maps were constructed based on the significantly differential metabolites (VIP ≥ 1, FC ≥ 1.2 or ≤ 0.8 and q-value < 0.05), which had a distinct segregation. There were 74 differential metabolites among the three groups in ESI+ mode (**Figure 4A**), and 80 in ESI− mode (**Figure 4B**), respectively.

**Figure 5:**
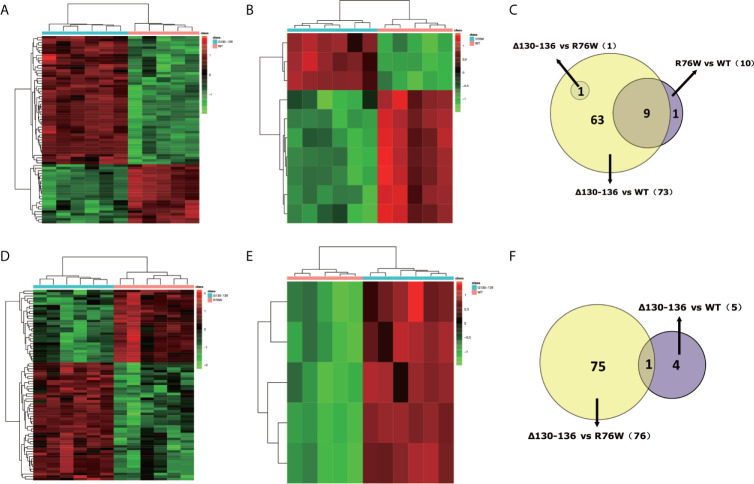
Discrimination of metabolic phenotype of primary osteocyte supernatants between Δ130-136, R76W and WT mice in ESI+ mode and ESI− mode. Heat maps of differential metabolites for Δ130-136 versus WT **(A)** and R76W versus WT **(B)** in ESI+ mode. Heat maps of differential metabolites for Δ130-136 versus R76W **(D)** and Δ130-136 versus WT **(E)** in ESI− mode. Venn diagrams exhibit the number of differential metabolites between these comparison groups in ESI+ mode **(C)** and ESI− mode **(F)**.

Besides, we constructed heat maps based on the differential metabolites to perform pairwise comparisons between the three mouse groups in ESI+ mode and ESI− mode. In ESI+ mode, 73 differential metabolites (50 metabolites were up-regulated, 23 metabolites were down-regulated) were found between Δ130-136 and WT mice (**Figure 5A**). In addition, there were 10 differential metabolites (3 metabolites were up-regulated, 7 metabolites were down-regulated) between R76W and WT mice (**Figure 5B**). However, there was only 1 differential metabolite between Δ130-136 and R76W mice, therefore no heat map was constructed between these two groups. As shown in **Figure 5C**, 9 overlapping differential metabolites were detected between Δ130-136 versus WT mice and R76W versus WT mice in ESI+ mode.

Likewise, we found 76 differential metabolites (47 metabolites were up-regulated, 29 metabolites were down-regulated) between Δ130-136 and R76W mice (**Figure 5D**) and 5 up-regulated metabolites between Δ130-136 and WT mice in ESI− mode (**Figure 5E**). Nevertheless, heat map did not exist between R76W and WT mice because there was no any differential metabolite between these two groups in ESI− mode. **Figure 5F** indicated that there was only 1 common differential metabolite between Δ130-136 versus R76W mice and Δ130-136 versus WT mice.

**Figure 6:**
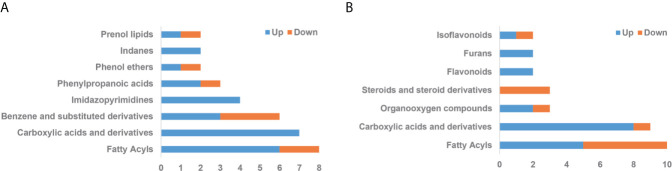
Categories of differential metabolites. **(A)** Δ130-136 versus WT in ESI+ mode. **(B)** Δ130-136 versus R76W in ESI− mode. X-axis represents the number of differential metabolites and y-axis represents chemical classes. Blue and jacinth indicate up-regulation and down-regulation, respectively.

By searching against mass-based HMDB/KEGG databases, we further discovered putative identifications for 58 differential metabolites between Δ130-136 and WT mice (**Table S1**) and 53 differential metabolites between Δ130-136 and R76W mice (**Table S2**) in ESI+ and ESI− mode, respectively. According to different biochemical properties, differential metabolites were further selected and divided into several categories between Δ130-136 and WT mice in ESI+ mode (**Table S3**) and between Δ130-136 and R76W mice in ESI− mode (**Table S4**). The top three categories identified between Δ130-136 and WT mice in ESI+ mode were “fatty acyls”, “carboxylic acids and derivatives”, and “benzene and substituted derivatives” (**Figure 6A**). As shown in **Table S3**, 7 differential metabolites belonging to carboxylic acids and derivatives were all up-regulated in Δ130-136 compared with WT mice, including cis-aconitic acid, phenylacetylglycine, and lyratol acetate. Similarly, “fatty acyls” and “carboxylic acids and derivatives” were also the most enriched differential metabolite categories between Δ130-136 and R76W mice (**Figure 6B**). Only one metabolite belonging to carboxylic acids and derivatives, L-glutamine, was down-regulated in Δ130-136 compared with R76W mice in ESI- mode (**Table S4**).

**Figure 7:**
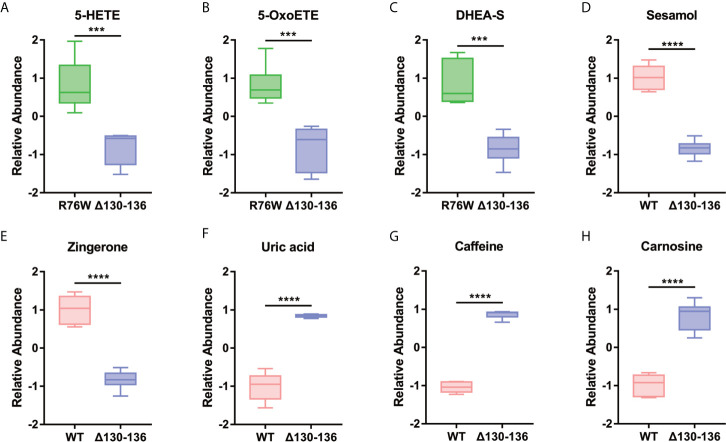
Representative differential metabolites related to the inflammatory reaction **(A**, **B)**, oxidative stress **(C**–**E)**, and musculoskeletal health **(F–H)** in the different groups. Data are mean ± SD; n=5-6; ***p < 0.001, ****p < 0.0001; t-test.

On the other hand, based on the different biological functions, we further selected potential differential metabolites with a close relationship with the function of Cx43 channels. As shown in **Figure 7**, we found two pro-inflammatory factors, 5-HETE and 5-OxoETE, were decreased in Δ130-136 as compared to R76W mice. Besides, the levels of several identified metabolites related to oxidative stress and musculoskeletal health were also significantly changed, including dehydroepiandrosterone sulfate (DHEA-S), sesamol (SES), zingerone (ZGR), uric acid (UA), caffeine, and carnosine. These results suggested that the differential metabolites may be the potentially released molecules regulated by Cx43 channels.

### Metabolic Pathway Analysis

**Figure 8:**
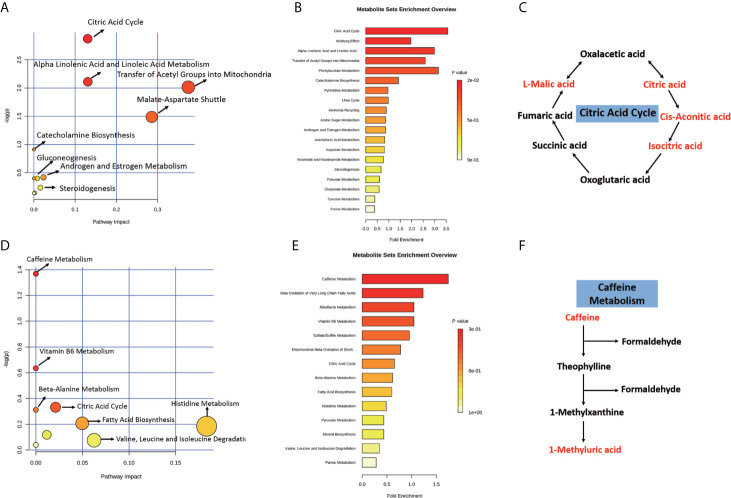
Pathway and enrichment analysis of the differential metabolites. With MetaboAnalyst 4.0, pathway and enrichment analysis were performed for Δ130-136 versus R76W in ESI− mode **(A, B)** and Δ130-136 versus WT in ESI+ mode **(D, E)**. Major enriched metabolic pathways including citric acid cycle **(C)** and caffeine metabolism **(F)**. Red characters indicate up-regulated metabolites.

To further elucidate the biological function of the differential metabolites, we performed metabolic pathway and enrichment analysis using MetaboAnalyst. As seen in **Figure 8A**, the most significantly affected pathways with lower p-values and higher pathway impact were “citric acid cycle”, “alpha linolenic acid and linoleic acid metabolism”, “transfer of acetyl groups into mitochondria, and “malate-aspartate shuttle” between Δ130-136 and R76W mice in ESI− mode. Consistent with the pathway analysis, enrichment analysis indicated that “citric acid cycle”, “alpha linolenic acid and linoleic acid metabolism”, and “transfer of acetyl groups into mitochondria” in Δ130-136 mice were significantly perturbed compared with that of R76W mice in ESI− mode (**Figure 8B**). More importantly, we found citric acid, cis-aconitic acid, isocitric acid, and L-malic acid were all obviously up-regulated in citric acid cycle in Δ130-136 mice compared with WT mice (**Figure 8C**). Moreover, “caffeine metabolism”, “vitamin B6 metabolism”, and “citric acid cycle” were the obviously distinct metabolic pathways between Δ130-136 and WT mice in ESI+ mode (**Figure 8D**). For enrichment analysis, caffeine metabolism was the most obviously affected (**Figure 8E**) and the levels of caffeine and 1-methyluric acid were also increased in Δ130-136 mice compared with that in WT mice (**Figure 8F**). The results indicated that “citric acid cycle” and “caffeine metabolism” may be closely related to the function of Cx43 channels.

## Discussion

Emerging data support the notion that bone acts as an endocrine organ and can produce signaling molecules to directly modulate metabolism of bone itself and distant target organs through feedforward and feedback regulatory loops (26). In osteocytes, Cx43 channels participate in regulating global metabolism through mediating the release of small molecules, such as ATP, IP3, PGE2, glutathione, amino acids and cyclic nucleotides (27–29). However, more secreted molecules remain to be identified, and the successful identification of unknown secreted molecules will be helpful to find some new biomarkers to explore the function of osteocytes and Cx43 channels. In this study, metabolomics analysis revealed several novel categories of molecules released through Cx43 channels, especially “fatty acyls” and “carboxylic acids and derivatives”. These metabolites may be candidate regulatory factors produced by osteocytes.

Among the potential metabolites, those related to inflammatory reaction and oxidative stress were of great interest given their close functional relation with Cx43 channels and osteocytes (**Figure 7**).

It has been demonstrated that osteocytes are implicated in inflammatory diseases such as rheumatoid arthritis (30) and may produce interleukin (IL)-1β, IL-6, and tumor necrosis factor-α (TNF-α) to mediate bone destruction in rheumatoid arthritis pathogenesis (31). In the current study, we also found two pro-inflammatory factors, 5-HETE and 5-OxoETE, were decreased in primary osteocytes of Δ130-136 compared with those in R76W mice, which was consistent with some previous studies regarding the roles of Cx43 channels in inflammatory responses (32). It was reported that blocking of Cx43 channels could reduce the release of proinflammatory cytokines in serum (33) and Cx43 hemichannel blocked by Peptide5 could protect against proinflammatory cytokines release and inhibit inflammasome activation (34, 35). Previous studies have indicated that 5-HETE and 5-OxoETE participated in the arachidonic acid (AA) metabolism as proinflammatory mediators (36–38). As reported, AA could not only be metabolized into prostaglandins (PGD2, PGE2, PGF2, PGH2, and PGI2) by COX-1/2 (36), but also transformed into 5-HETE and 5-OxoETE by 5-lipoxygenase or 5-hydroxyeicosanoid dehydrogenase under cellular stimulation. Furthermore, opening of Cx43 hemichannels resulted in the increased release of PGE2, which may be partly attributed to the increased expression of AA. That may be the reason of increased proinflammatory molecules 5-HETE and 5-OxoETE in R76W mice (hemichannel opening is promoted). Taken together, Cx43 channels may show some effect in the osteocyte-mediated inflammatory process and hemichannel promotion could inhibit the inflammatory cascade *via* decreasing the release of proinflammatory molecules.

n our study, we also found some differential metabolites associated with oxidative stress, including dehydroepiandrosterone sulfate (DHEA-S), sesamol (SES), and zingerone (ZGR). A recent study has demonstrated that connexin hemichannels can protect lens fiber cells against cell death induced by oxidative stress (39). Additionally, H_2_O_2_ could open Cx43 hemichannels to protect osteocytes from oxidative stress-induced apoptosis. Osteocytes were more susceptible to H_2_O_2_ induced apoptosis when they were treated with Cx43 (E2) antibody to specifically block Cx43 hemichannel activity (40). Dehydroepiandrosterone (DHEA) and DHEA-S can mutually transform into each other (41), and DHEA has been shown to prevent oxidative stress *in vivo* and *in vitro* (42–44). As such, the promotion of Cx43 hemichannels in R76W mice may facilitate the release of DHEA-S, which may be related to the response to oxidative stress. In addition, Δ130-136 mice showed decrease of antioxidative molecules SES and ZGR compared to WT mice. SES attenuated H_2_O_2_-induced oxidative stress and potentially protected neuronal cells (45). ZGR showed nephroprotective effects in rat model of nephrotoxicity mostly through suppression of oxidative stress (46). Our results indicated that promotion of Cx43 hemichannels increased the release of SES and ZGR, which seemed to enhance the protective effect against oxidative stress. That provided clues to further investigate their roles in osteocytes.

Interestingly, we also detected higher expression of uric acid (UA), caffeine and carnosine in Δ130-136 mice compared with WT mice. UA is the final product of purine metabolism, which could play essential roles in diverse physiological processes, especially in bone health (47). Besides, caffeine, a kind of purine alkaloid, is involved in numerous biological reactions, such as lipid metabolism and muscle relaxation (48, 49). Carnosine mainly serves as a neuroprotective dipeptide (50, 51). The findings that Δ130-136 mice leaded to higher expression of these molecules indicated close association between Cx43 channels and various biological functions. We hypothesized that when Cx43 hemichannel was blocked, additional channels may increase permeability to cause the elevated levels of related molecules, which could form a protective mechanism under different pathophysiological conditions. But the accurate regulation mechanisms still need to be further explored.

Finally, metabolic pathway analysis pointed out that the citric acid cycle was obviously changed among mice of disparate Cx43 channel function. L-malic acid, citric acid, cis-aconitic acid, and isocratic acid were the greatest changed metabolites. Citric acid cycle was fully elucidated to be implicated in energy metabolism and a key part of the process of ATP production. It has been well described that ATP release occurred through Cx43-formed hemichannels in many cell types. Interestingly, a recent study has indicated that lack or inhibition of Cx43 might affect mitochondrial respiration and mitochondrial ATP-production (52). It is likely that Cx43 channels have an impact on ATP release by disturbing citric acid cycle, which may further affect the global energy metabolism. Therefore, it is worth to investigate the roles of Cx43 channels in citric acid cycle and mitochondrial respiration.

Osteocytes are known to be the major mechanosensory cells in bone tissue (53). One of the most essential biological functions of osteocytes is to sense the mechanical stimulation, translate and transmit chemical signals to adjacent bone cells and other tissue cells, which is mainly mediated by Cx43 channels (54, 55). Currently, most studies have focused on exploring the function of Cx43 channels in response to mechanical loading (56, 57). However, in the present study, the primary osteocytes we used were in the static condition, which could be considered as a form of unloading. We found Cx43 channels in osteocytes could still react to mechanical unloading by regulating the release of related molecules. Furthermore, our previous study has also elucidated the function of Cx43 channels in response to mechanical unloading *in vivo*, which indicates that inhibiting osteocytic Cx43 channels promotes bone loss induced by unloading. Our next step is to investigate the function of Cx43 channels in primary osteocytes under mechanical or chemical stimulation *in vitro*, which will aid to further understand the roles of Cx43 channels.

In conclusion, our study has identified the changes in the metabolic profiles of primary osteocyte supernatants of WT, R76W and Δ130-136 mice by a non-targeted metabolomics method. Several new categories of molecules released through Cx43 channels were found, especially “fatty acyls” and “carboxylic acids and derivatives”. Alteration of Cx43 channel function could affect the release of metabolites related to inflammatory reaction, including 5-HETE and 5-OxoETE, and oxidative stress such as DHEA-S, SES and ZGR. Additionally, citric acid cycle was also significantly affected while Cx43 channel function was changed. These findings provided some effective clues to better understand the function of Cx43 channels, and suggested Cx43 could be a new therapeutic target to treat bone-related diseases. The function of new-found differential metabolites will be further investigated in osteocytes in our future study.

## Data Availability Statement

The datasets presented in this study can be found in online repositories. The names of the repository/repositories and accession number(s) can be found below: https://www.ebi.ac.uk/metabolights/MTBLS2358.

## Ethics Statement

The animal study was reviewed and approved by Northwestern Polytechnical University (NPU) Institutional Animal Care and Use Committee.

## Author Contributions

HX, D-EW and JJ contributed to the conception and design of the study, prepared the manuscript and made a revision of the manuscript. MC and LZ designed the study, conducted the experiment, analyzed the data, drafted and revised the manuscript. GL, KN, and BY analyzed the data. All authors contributed to the article and approved the submitted version.

## Funding

This research was funded by National Natural Science Foundation of China (Nos. 81772409 and 81472090), Space Medical Experiment Project of China Manned Space Program (No: HYZHXM01024), National Institutes of Health (No. AG045040) and Welch Foundation (No. AQ-1507).

## Conflict of Interest

The authors declare that the research was conducted in the absence of any commercial or financial relationships that could be construed as a potential conflict of interest.
