# Supplementary material for: Primary Osteocyte Supernatants Metabolomic Profiling of Two Transgenic Mice With Connexin43 Dominant Negative Mutants
Source: Front Endocrinol (Lausanne). 2021 May 18;12:649994. doi: 10.3389/fendo.2021.649994 (PMC8169970; doi:10.3389/fendo.2021.649994)
Supplement: Supplementary file 1 [file DataSheet_1.pdf]

Supplementary Materials

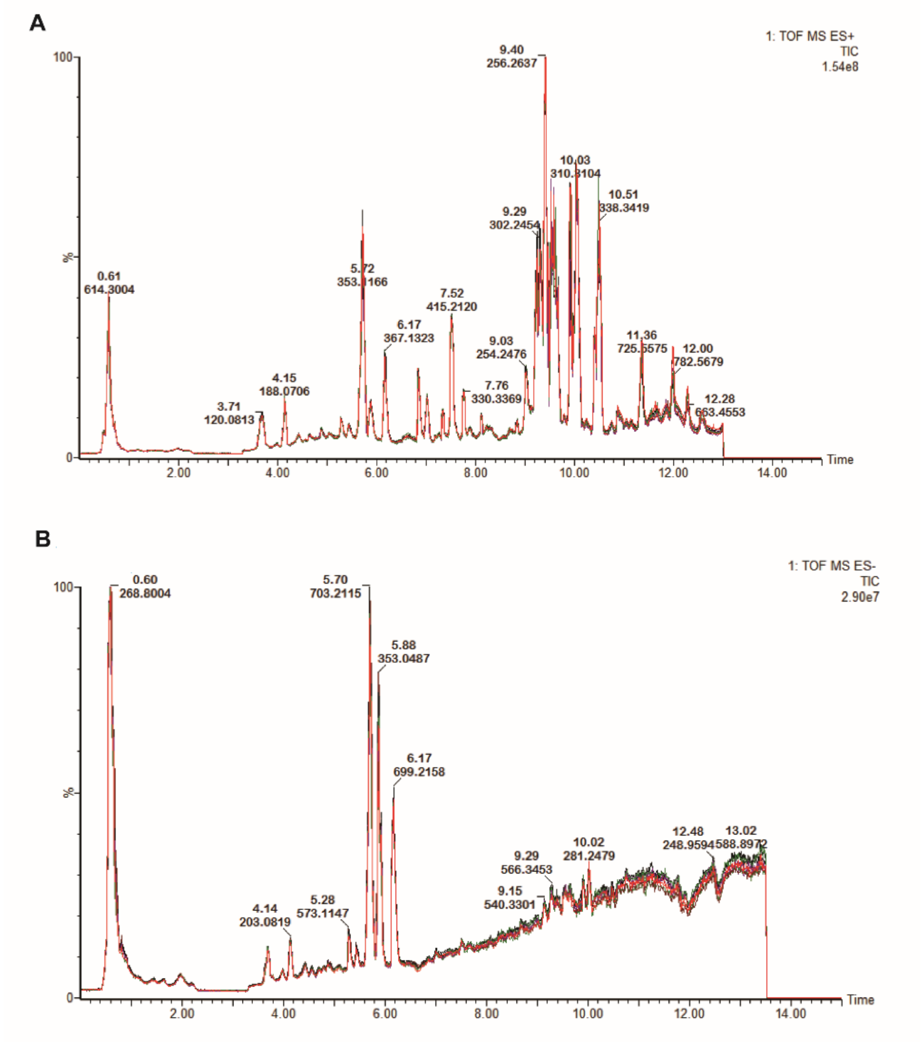

Supplementary Figure 1. Overlay of all total ion current (TIC) chromatograms of quality control (QC) samples in ESI+ mode (A) and ESI− mode (B). x- and y-axis represent the time and intensity, respectively.

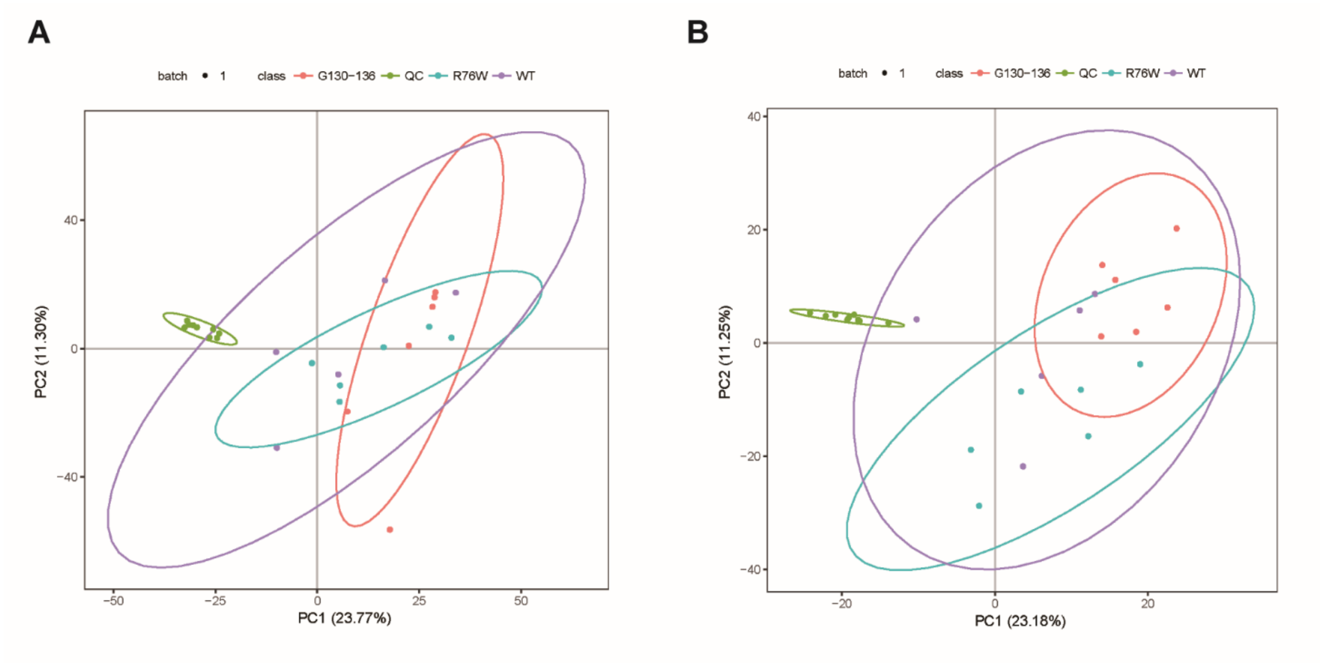

Supplementary Figure 2. PCA score scatter plots of quality control (QC) and tested samples. PCA score scatter plots in ESI+ mode (A) and ESI− mode (B). QC (green), n=9; WT (purple), n=5; R76W (blue), n=6; Δ130-136 (red), n=6.

Supplementary Table 1. List of differential metabolites between Δ130-136 and WT mice in ESI+ mode.

MS (m/z)	RT (min)	VIP	q-value	Ratio	Description	HMDB or KEGG ID
330.1037	0.7265	2.098445	0.043189	1.463448	Inosine-5'-carboxylate	C03280
428.0716	0.7265	1.966165	0.043189	1.300047	Hetacillin potassium	C13980
280.1403	1.376017	1.704629	0.027344	1.257856	N-(1-Deoxy-1-fructosyl) valine	HMDB0037844
151.0431	2.409917	4.921188	0.008883	7.408306	(±)-2-Hydroxy-4-(methylthio)butanoic acid	HMDB37115
530.2284	3.429567	2.488674	0.028711	1.9116	Darunavir	HMDB0015393
231.0838	3.856767	3.07593	0.008442	2.162822	Carbapenem biosynthesis intermediate 1	C20817
186.0285	5.83615	5.578995	0.006632	8.643898	L-Albizziine	C08264
388.0616	0.712217	1.916488	0.049654	1.375479	10-Hydroxydihydrosanguinarine	C05247
340.1097	0.7265	2.425134	0.039835	1.507886	Rosiglitazone	HMDB0005031
229.1191	1.012233	3.195338	0.009952	1.819804	Methyldopa	HMDB0011754
201.0876	1.23315	2.356524	0.018237	1.552773	4-Pyridoxic acid	HMDB0000017
190.0713	2.43065	5.05588	0.043189	9.27569	Dihydrolipoamide	HMDB0000985
192.0508	2.444933	4.92597	0.020029	7.74849	cis-Aconitic acid	HMDB0000072
151.0247	2.459217	5.416831	0.036173	12.96493	Uric acid	HMDB0000289
165.0398	2.4735	4.456463	0.016205	5.455922	1-Methyluric acid	HMDB0003099
211.1076	3.78245	1.796537	0.008005	1.321933	Phenylacetylglycine	HMDB0000821
190.0871	3.885333	1.995611	0.007766	1.306953	N-Acetyl-L-phenylalanine	HMDB0000512
200.0708	4.056767	2.760962	0.003216	1.72538	3-Methylindolepyruvate	C05644
157.0825	4.206083	3.314036	0.005647	2.247355	Polypropylene glycol (m w 1,200-3,000)	HMDB0032478
219.0544	4.220367	2.774578	0.013729	1.999358	(4-Ethoxyphenyl) urea	HMDB0032565
174.0549	4.755617	2.889462	0.011639	1.648433	1-nitronaphthalene	HMDB0062188
377.1455	4.89065	3.495501	0.03236	0.400448	Riboflavin	HMDB0000244
273.1244	5.151383	3.079533	0.023591	1.792724	9-Pentadecene-12,14-diyne-1,11-diol	HMDB0034562

315.109	5.340383	2.550735	0.038701	1.463593	Vanilloside	HMDB0029664
374.1144	5.503983	2.992482	0.049654	1.880755	Deferasirox	HMDB0015547
263.0557	5.804	3.419838	0.009585	2.246997	Maclurin	HMDB0032644
183.05	5.83615	3.83945	0.002885	3.211203	7-Methyluric acid	HMDB0011107
195.0885	5.83615	3.897915	0.000131	3.104221	Caffeine	C07481
179.0554	5.8533	4.493916	0.018237	4.903733	Galactonic acid	HMDB0000565
275.1011	5.867583	1.982793	0.021503	1.394214	1,3,5-Trihydroxy-10-methylacridone	HMDB0041468
265.0685	5.867583	2.695132	0.004836	1.607361	Carnosine	HMDB0000033
239.0566	5.881867	3.476609	0.000131	2.584109	(2R,3S)-Piscidic acid	HMDB0030809
247.0405	5.881867	1.683318	0.041882	0.820964	(1R,2R)-3-[(1,2-Dihydro-2-hydroxy-1-naphthalenyl)thio]-2-oxopropanoic acid	C14799
392.0317	5.881867	2.270488	0.049798	0.663962	Adenosine 5'-phosphate disodium	C18344
249.1093	5.924733	2.736666	0.023591	1.970599	N2-Succinyl-L-glutamic acid 5-semialdehyde	HMDB0001180
255.0527	5.996167	1.659461	0.005239	1.312668	Sampangine	HMDB0034291
225.0432	6.01045	4.300983	0.009585	3.841853	1-Methoxy-1H-indole-3-acetonitrile	HMDB0040973
256.0418	6.259767	3.207871	0.004836	2.270425	Captopril	HMDB0015328
235.1324	6.915667	3.429004	0.001667	2.531336	(10S,11S)-Pterosin C	HMDB0030763
233.1172	7.293567	2.141416	0.039084	1.775672	(S, Z)-Lyratol acetate	HMDB0031840
219.1374	7.30785	2.341504	0.020507	1.522402	(R)-Pterosin B	HMDB0030759
109.0652	7.450983	2.744821	0.015109	0.551709	Allyl crotonate	HMDB0032067
121.0287	7.450983	3.082364	0.00556	0.410739	Sesamol	HMDB0033812
153.0548	7.450983	2.917534	0.017089	0.477646	3-Methoxybenzoic acid	HMDB0032606
167.0704	7.450983	2.986602	0.006978	0.411815	D-Phenyllactic acid	HMDB0000563
167.1048	7.450983	3.53421	0.011474	0.338556	Caprylic acid	HMDB0000482
169.0755	7.450983	2.688193	0.00556	0.501871	Beta-Carboline	HMDB0012897

181.086	7.450983	2.971924	0.004836	0.413066	2-Amino-6-methyldipyrido[1,2-a:3',2'-d] imidazole	HMDB0029749
195.1017	7.450983	2.874196	0.009585	0.429431	Zingerone	HMDB0032590
297.0641	7.450983	1.858818	0.016001	0.611806	Geranyl-PP	HMDB0001285
123.0807	7.465267	2.775795	0.020507	0.528787	1-Phenylethanol	HMDB0032619
95.04946	7.465267	3.073141	0.00556	0.418256	Phenol	HMDB0000228
251.1265	7.657433	2.802749	0.010173	1.669588	Traumatic acid	HMDB0000933
233.1168	7.771733	4.032979	0.001667	3.915656	Jasmonic acid	HMDB0032797
225.1458	7.90675	2.780619	0.015109	1.731778	3-Hydroxynonyl acetate	HMDB0032443
265.1434	8.149617	3.245622	0.018237	2.455942	(S)-Abscisic acid	HMDB0035140
203.143	9.0692	3.262401	0.00556	0.387155	Feniculin	HMDB0030838
371.1008	9.7538	3.419578	0.005984	0.349203	Zopiclone	HMDB0015329

RT: Retention time; VIP: variable importance for the projection.

Supplementary Table 2. List of differential metabolites between Δ130-136 and R76W mice in ESI- mode.

MS (m/z)	RT (min)	VIP	q-value	Ratio	Description	HMDB or KEGG ID
300.1192	0.724417	2.3459	0.037492	1.682028	Esprocarb	C14526
368.0042	0.752983	1.630096	0.037492	1.365125	Eudistomin C	C17167
403.0188	1.053017	1.607497	0.045033	1.314396	Spiro[benzofuran-2(3H),1'-[2]cyclohexene]-7-chloro-4,6-dimethoxy-6'-methyl-2'- (methylthio)-3,4'-dione	C15300
585.486	10.02423	2.96467	0.049502	0.430422	Erythrinasinate A	HMDB0038713
416.1774	3.6032	1.403203	0.045852	1.381871	Tryprostatin A	C20607
149.0271	4.127483	2.654674	0.022669	1.799783	(±)-2-Hydroxy-4-(methylthio)butanoic acid	HMDB0037115
381.0365	0.525733	2.268286	0.037492	1.575189	Limocitrin	HMDB0029516
145.0613	0.6277	1.852454	0.045292	0.734934	L-Glutamine	HMDB0000641
133.0136	0.724417	2.021082	0.037492	1.445709	L-Malate	C00149
185.056	0.724417	2.305998	0.045292	1.506752	Pyroglutamylglycine	HMDB0061890
259.0932	0.724417	2.708225	0.037492	1.868771	Glutamyl-Hydroxyproline	HMDB0028820
342.0835	0.724417	2.782111	0.043005	1.811296	DIBOA-Glc	HMDB0033734
111.0082	0.7387	2.124636	0.037492	1.483692	2-Furoic acid	HMDB0000617
115.003	0.7387	1.900365	0.037492	1.405848	Maleic acid	HMDB0000176
173.0083	0.7387	2.235655	0.022669	1.492887	Dehydroascorbic acid	HMDB0001264
191.019	0.7387	1.98539	0.037492	1.417319	Diketogulonic acid	HMDB0005971
284.1246	0.7387	3.241007	0.037492	2.340127	Glycylprolylhydroxyproline	HMDB0002171
381.0302	0.7387	1.365509	0.038277	1.238019	hesperetin 3'-O-sulfate	HMDB0029202
129.0188	0.752983	1.652276	0.045292	1.298427	Gamma-delta-Dioxovaleric acid	HMDB0013233
403.0215	0.752983	1.197094	0.037492	1.249838	5-(acetyloxy)-13-hydroxy-9-oxo-8,17-dioxatetracyclo[8.7.0.0^{2},^{7}.0^{11},^{16}] heptadeca-1(10),2(7),3,5,11,13,15-heptaen-14-yl acetate	HMDB0128402

325.014	0.91015	1.961776	0.039401	1.412531	4-Hydroxy-5-(4'-hydroxyphenyl)-valeric acid-4'-O-sulphate	HMDB0059976
147.0293	1.31995	1.510578	0.037492	1.242099	Citramalic acid	HMDB0000426
111.0081	1.41545	2.210009	0.037492	1.511959	3-Furoic acid	HMDB0000444
129.0186	1.41545	1.824275	0.043031	1.340527	Mesaconic acid	HMDB0000749
173.0084	1.41545	2.316645	0.034578	1.534167	cis-Aconitic acid	HMDB0000072
191.0189	1.41545	2.109432	0.037492	1.473907	Isocitric acid	HMDB0000193
185.0561	1.544033	3.093982	0.043031	2.177576	AMPA	C13672
440.0381	1.644033	1.580783	0.037492	0.788643	Tizoxanide glucuronide	HMDB0060643
129.0184	1.6726	1.889306	0.049166	1.394336	Mesaconate	C01732
284.1242	1.6869	3.437816	0.037492	2.363225	Isothipendyl	HMDB0015692
349.2353	10.02423	2.178406	0.037492	0.657541	Tetrahydrocorticosterone	C05476
417.2225	10.02423	2.290912	0.037492	0.62842	Apo-12'-violaxanthal	HMDB0034953
553.1976	10.02423	2.131651	0.037492	0.672838	Marmesin rutinoside	HMDB0041413
621.1848	10.02423	2.02114	0.037492	0.695526	2-{2,6-dihydroxy-4-[6-hydroxy-7-(3-methylbut-2-en-1-yl)-1- benzofuran-2-yl]-3-methoxyphenyl}-6-(2,4-dihydroxyphenyl)- 4-methylcyclohex-3-ene-1-carboxylic acid	HMDB0126228
331.2632	10.07687	4.293577	0.037492	0.219512	Adrenic acid	HMDB0002226
191.0191	3.321967	1.638063	0.045292	1.287251	Citric acid	HMDB0000094
241.0819	3.35055	3.002741	0.022669	2.215581	Thymidine	HMDB0000273
346.1067	3.941767	2.94679	0.049502	1.982687	Domoic acid	HMDB0033939
259.0748	4.237267	3.141338	0.037492	2.073062	Diplosporin	HMDB0030680
367.0645	5.790883	1.301988	0.037492	1.279153	Sanguinarine	HMDB0029367
315.2529	8.131483	1.223601	0.049166	0.729235	MG (15:0/0:0/0:0)	HMDB0011563
317.2112	8.326983	2.528013	0.037492	0.56353	5-OxoETE	C14732
319.2269	8.498433	1.958763	0.043005	0.682454	5-HETE	HMDB0011134
387.2139	8.512717	2.256588	0.037492	0.590498	3-keto-Digoxigenin	HMDB0060746

343.2269	8.58415	1.798209	0.037492	0.721648	Medroxyprogesterone	HMDB0001939
411.2144	8.58415	1.973154	0.037492	0.664213	Austalide K	HMDB0030157
345.2426	8.751083	1.974646	0.037492	0.692933	Ginkgoic acid	HMDB0033897
293.2117	8.79395	2.499365	0.037492	0.557071	9-OxoODE	HMDB0004669
249.185	9.018333	3.91803	0.032396	0.20985	Norambreinolide	HMDB0035293
478.293	9.2755	1.842865	0.037492	1.348671	LysoPE(18:1(9Z)/0:0)	HMDB0011506
377.1413	9.41385	2.599977	0.037492	0.514035	Demethylcalabaxanthone	HMDB0030656
277.2163	9.42815	2.63329	0.041869	0.512934	Alpha-Linolenic acid	HMDB0001388
367.157	9.7808	3.635551	0.037492	0.298281	Dehydroepiandrosterone sulfate	HMDB0001032

RT: Retention time; VIP: variable importance for the projection.

Supplementary Table 3. List of differential metabolites of different categories between Δ130-136 and WT mice in ESI+ mode.

MS (m/z)	RT (min)	VIP	q-value	Ratio	Description	Class	HMDB or KEGG ID
151.0431	2.409917	4.921188	0.008883	7.408306	(±)-2-Hydroxy-4-(methylthio)butanoic acid	Fatty Acyls	HMDB37115
190.0713	2.43065	5.05588	0.043189	9.27569	Dihydrolipoamide	Fatty Acyls	HMDB0000985
273.1244	5.151383	3.079533	0.023591	1.792724	9-Pentadecene-12,14-diyne-1,11-diol	Fatty Acyls	HMDB0034562
109.0652	7.450983	2.744821	0.015109	0.551709	Allyl crotonate	Fatty Acyls	HMDB0032067
167.1048	7.450983	3.53421	0.011474	0.338556	Caprylic acid	Fatty Acyls	HMDB0000482
251.1265	7.657433	2.802749	0.010173	1.669588	Traumatic acid	Fatty Acyls	HMDB0000933
233.1168	7.771733	4.032979	0.001667	3.915656	Jasmonic acid	Fatty Acyls	HMDB0032797
225.1458	7.90675	2.780619	0.015109	1.731778	3-Hydroxynonyl acetate	Fatty Acyls	HMDB0032443
280.1403	1.376017	1.704629	0.027344	1.257856	N-(1-Deoxy-1-fructosyl) valine	Carboxylic acids and derivatives	HMDB0037844
192.0508	2.444933	4.92597	0.020029	7.74849	cis-Aconitic acid	Carboxylic acids and derivatives	HMDB0000072
211.1076	3.78245	1.796537	0.008005	1.321933	Phenylacetylglycine	Carboxylic acids and derivatives	HMDB0000821
190.0871	3.885333	1.995611	0.007766	1.306953	N-Acetyl-L-phenylalanine	Carboxylic acids and derivatives	HMDB0000512
249.1093	5.924733	2.736666	0.023591	1.970599	N2-Succinyl-L-glutamic acid 5-semialdehyde	Carboxylic acids and derivatives	HMDB0001180
256.0418	6.259767	3.207871	0.004836	2.270425	Captopril	Carboxylic acids and derivatives	HMDB0015328
233.1172	7.293567	2.141416	0.039084	1.775672	(S, Z)-Lyratol acetate	Carboxylic acids and derivatives	HMDB0031840
530.2284	3.429567	2.488674	0.028711	1.9116	Darunavir	Benzene and substituted derivatives	HMDB0015393
219.0544	4.220367	2.774578	0.013729	1.999358	(4-Ethoxyphenyl) urea	Benzene and substituted derivatives	HMDB0032565
263.0557	5.804	3.419838	0.009585	2.246997	Maclurin	Benzene and substituted derivatives	HMDB0032644
153.0548	7.450983	2.917534	0.017089	0.477646	3-Methoxybenzoic acid	Benzene and substituted derivatives	HMDB0032606
195.1017	7.450983	2.874196	0.009585	0.429431	Zingerone	Benzene and substituted derivatives	HMDB0032590
123.0807	7.465267	2.775795	0.020507	0.528787	1-Phenylethanol	Benzene and substituted derivatives	HMDB0032619
151.0247	2.459217	5.416831	0.036173	12.96493	Uric acid	Imidazopyrimidines	HMDB0000289
165.0398	2.4735	4.456463	0.016205	5.455922	1-Methyluric acid	Imidazopyrimidines	HMDB0003099
183.05	5.83615	3.83945	0.002885	3.211203	7-Methyluric acid	Imidazopyrimidines	HMDB0011107

195.0885	5.83615	3.897915	0.000131	3.104221	Caffeine	Imidazopyrimidines	C07481
229.1191	1.012233	3.195338	0.009952	1.819804	Methyldopa	Phenylpropanoic acids	HMDB0011754
239.0566	5.881867	3.476609	0.000131	2.584109	(2R,3S)-Piscidic acid	Phenylpropanoic acids	HMDB0030809
167.0704	7.450983	2.986602	0.006978	0.411815	D-Phenyllactic acid	Phenylpropanoic acids	HMDB0000563
340.1097	0.7265	2.425134	0.039835	1.507886	Rosiglitazone	Phenol ethers	HMDB0005031
203.143	9.0692	3.262401	0.00556	0.387155	Feniculin	Phenol ethers	HMDB0030838
235.1324	6.915667	3.429004	0.001667	2.531336	(10S,11S)-Pterosin C	Indanes	HMDB0030763
219.1374	7.30785	2.341504	0.020507	1.522402	(R)-Pterosin B	Indanes	HMDB0030759
297.0641	7.450983	1.858818	0.016001	0.611806	Geranyl-PP	Prenol lipids	HMDB0001285
265.1434	8.149617	3.245622	0.018237	2.455942	(S)-Abscisic acid	Prenol lipids	HMDB0035140

RT: Retention time; VIP: variable importance for the projection.

Supplementary Table 4. List of differential metabolites of different categories between Δ130-136 and R76W mice in ESI- mode.

MS (m/z)	RT (min)	VIP	q-value	Ratio	Description	Class	HMDB or KEGG ID
149.0271	4.127483	2.654674	0.022669	1.799783	(±)-2-Hydroxy-4-(methylthio)butanoic acid	Fatty Acyls	HMDB0037115
325.014	0.91015	1.961776	0.039401	1.412531	4-Hydroxy-5-(4'-hydroxyphenyl)-valeric acid-4'-O-sulphate	Fatty Acyls	HMDB0059976
147.0293	1.31995	1.510578	0.037492	1.242099	Citramalic acid	Fatty Acyls	HMDB0000426
129.0186	1.41545	1.824275	0.043031	1.340527	Mesaconic acid	Fatty Acyls	HMDB0000749
129.0184	1.6726	1.889306	0.049166	1.394336	Mesaconate	Fatty Acyls	C01732
331.2632	10.07687	4.293577	0.037492	0.219512	Adrenic acid	Fatty Acyls	HMDB0002226
317.2112	8.326983	2.528013	0.037492	0.56353	5-OxoETE	Fatty Acyls	C14732
319.2269	8.498433	1.958763	0.043005	0.682454	5-HETE	Fatty Acyls	HMDB0011134
293.2117	8.79395	2.499365	0.037492	0.557071	9-OxoODE	Fatty Acyls	HMDB0004669
277.2163	9.42815	2.63329	0.041869	0.512934	Alpha-Linolenic acid	Fatty Acyls	HMDB0001388
145.0613	0.6277	1.852454	0.045292	0.734934	L-Glutamine	Carboxylic acids and derivatives	HMDB0000641
185.056	0.724417	2.305998	0.045292	1.506752	Pyroglutamylglycine	Carboxylic acids and derivatives	HMDB0061890
259.0932	0.724417	2.708225	0.037492	1.868771	Glutamyl-Hydroxyproline	Carboxylic acids and derivatives	HMDB0028820
115.003	0.7387	1.900365	0.037492	1.405848	Maleic acid	Carboxylic acids and derivatives	HMDB0000176
284.1246	0.7387	3.241007	0.037492	2.340127	Glycylprolylhydroxyproline	Carboxylic acids and derivatives	HMDB0002171
173.0084	1.41545	2.316645	0.034578	1.534167	cis-Aconitic acid	Carboxylic acids and derivatives	HMDB0000072
191.0189	1.41545	2.109432	0.037492	1.473907	Isocitric acid	Carboxylic acids and derivatives	HMDB0000193
191.0191	3.321967	1.638063	0.045292	1.287251	Citric acid	Carboxylic acids and derivatives	HMDB0000094
346.1067	3.941767	2.94679	0.049502	1.982687	Domoic acid	Carboxylic acids and derivatives	HMDB0033939
342.0835	0.724417	2.782111	0.043005	1.811296	DIBOA-Glc	Organooxygen compounds	HMDB0033734
191.019	0.7387	1.98539	0.037492	1.417319	Diketogulonic acid	Organooxygen compounds	HMDB0005971
440.0381	1.644033	1.580783	0.037492	0.788643	Tizoxanide glucuronide	Organooxygen compounds	HMDB0060643
387.2139	8.512717	2.256588	0.037492	0.590498	3-keto-Digoxigenin	Steroids and steroid derivatives	HMDB0060746
343.2269	8.58415	1.798209	0.037492	0.721648	Medroxyprogesterone	Steroids and steroid derivatives	HMDB0001939

367.157	9.7808	3.635551	0.037492	0.298281	Dehydroepiandrosterone sulfate	Steroids and steroid derivatives	HMDB0001032
381.0365	0.525733	2.268286	0.037492	1.575189	Limocitrin	Flavonoids	HMDB0029516
381.0302	0.7387	1.365509	0.038277	1.238019	hesperetin 3'-O-sulfate	Flavonoids	HMDB0029202
111.0082	0.7387	2.124636	0.037492	1.483692	2-Furoic acid	Furans	HMDB0000617
111.0081	1.41545	2.210009	0.037492	1.511959	3-Furoic acid	Furans	HMDB0000444
403.0215	0.752983	1.197094	0.037492	1.249838	5-(acetyloxy)-13-hydroxy-9-oxo-8,17-dioxatetracyclo[$8.7.0.0^{2,7}.0^{11,16}$] heptadeca-1(10),2(7),3,5,11,13,15-heptaen-14-yl acetate	Isoflavonoids	HMDB0128402
411.2144	8.58415	1.973154	0.037492	0.664213	Austalide K	Isoflavonoids	HMDB0030157

RT: Retention time; VIP: variable importance for the projection.
